# Classification of metabolic syndrome according to lipid alterations: analysis from the Mexican National Health and Nutrition Survey 2006

**DOI:** 10.1186/1471-2458-14-1056

**Published:** 2014-10-09

**Authors:** Andrea Pedroza-Tobias, Belem Trejo-Valdivia, Luz M Sanchez-Romero, Simon Barquera

**Affiliations:** Instituto Nacional de Salud Publica, Cuernavaca, Morelos México; Department of Epidemiology and Public Health, University College London, London, UK

**Keywords:** Metabolic syndrome, Dyslipidemias, Triglycerides, HDL cholesterol, Mexico

## Abstract

**Background:**

There are 16 possible Metabolic Syndrome (MS) combinations out of 5 conditions (glucose intolerance, low levels of high-density lipoprotein Cholesterol (HDL-C), high triglycerides, high blood pressure and abdominal obesity), when selecting those with at least three. Studies suggest that some combinations have different cardiovascular risk. However evaluation of all 16 combinations is complex and difficult to interpret. The purpose of this study is to describe and explore a classification of MS groups according to their lipid alterations.

**Methods:**

This is a cross-sectional study with data from the Mexican National Health and Nutrition Survey 2006. Subjects (n = 5,306) were evaluated for the presence of MS; four mutually-exclusive MS groups were considered: *mixed dyslipidemia* (altered triglycerides and HDL-C), *hypoalphalipoproteinemia*: (normal triglycerides but low HDL-C), *hypertriglyceridemia* (elevated triglycerides and normal HDL-C) and *without dyslipidemia* (normal triglycerides and HDL-C). A multinomial logistic regression model was fitted in order to identify characteristics that were associated with the groups.

**Results:**

The most frequent MS group was hypoalphalipoproteinemia in females (51.3%) and mixed dyslipidemia in males (43.5%). The most prevalent combination of MS for both genders was low HDL-C + hypertension + abdominal obesity (20.4% females, 19.4% males). The hypoalphalipoproteinemia group was characteristic of women and less developed areas of the country. The group without dyslipidemia was more frequent in the highest socioeconomic level and less prevalent in the south of the country. The mixed dyslipidemia group was characteristic of men, and the Mexico City region.

**Conclusions:**

A simple system to classify MS based on lipid alterations was useful to evaluate prevalences by diverse biologic and sociodemographic characteristics. This system may allow prevention and early detection strategies with emphasis on population-specific components and may serve as a guide for future studies on MS and cardiovascular risk.

## Background

In recent years, Mexico has undergone the so-called nutritional transition in which dietary and physical activity patterns have changed, bringing with it a change in chronic disease patterns and a decrease in infectious diseases [1]. However, the level of this transition varies depending on the geographical area of the country. Mexico’s southern region is the least developed region. It is characterized by a traditional diet and a higher level of physical activity, while the northern region is more developed and its population usually consumes an energy dense diet rich in sugar, fat and sodium [2].

Metabolic syndrome (MS) refers to a combination of metabolic risk factors that lead to cardiovascular events (CVE) [3, 4]. According to the most recent classification for MS, at least three of the following altered conditions are required in order to arrive at a diagnosis: high waist circumference, high blood pressure, high triglycerides, low high-density lipoprotein cholesterol (HDL-C) and high fasting glucose [4]. These criteria allow for sixteen possible combinations of MS that do not necessarily involve the same cardiovascular risk.

Some studies have identified combinations of components of MS that have a higher risk of CVE, cerebrovascular events and mortality [5–9]. Additionally, it has been shown that low HDL-C levels and high triglyceride levels are an independent risk factor for cardiovascular disease [10–12]. Moreover, it has been proposed to use the triglyceride/HDL-C ratio as a marker of cardiovascular risk and insulin resistance [12].

According to the 2006 Mexican National Health and Nutrition Survey (ENSANUT from its Spanish acronym), the prevalence of MS in Mexico approaches 49.8% (47.5%-52.1%) in the adult population (≥ 20 years old) [13]. Although 31.5% of Mexican adults have hypertriglyceridemia, only 3.8% of the population reported to been previously diagnosed, thus making this one of the main national challenges for public health [14].

This study focus on identifying and describing MS groups based on the presence of hypertriglyceridemia and/or hypoalphalipoproteinema since they play an important role in the development of MS, insulin resistance and cardiovascular risk, and are generally asymptomatic.

## Methods

A secondary analysis was carried out on data obtained from the ENSANUT 2006. This survey was conducted from October 2005 to May 2006. ENSANUT 2006 is a probabilistic, cluster-stratified survey with national, regional and state representation that collected health, nutrition and sociodemographic information from 48,600 households. In addition, fasting venous blood samples were obtained from a randomized subsample designed with adequate power to detect conditions with a prevalence of at least 8% at the national level, by region and urban/rural location, resulting in a total of 6,021 subjects ≥20 y [15]. The sampling weights were calculated considering the age and sex distribution of the Mexican National population census. No statistically significant differences were found in biological and socio-economic characteristics between the subsample and the whole adult ENSANUT 2006 sample [13]. The present analysis was conducted in 2012–2013.

### Data collection

Trained surveyors administered a standardized questionnaire to the subjects in order to obtain personal data such as age, level of education, socioeconomic level, alcohol and tobacco consumption, as well as chronic disease diagnostic and treatment information [16].

Anthropometric information was obtained using standard procedures by trained personnel. Waist circumference was measured at the midpoint between the highest part of the iliac crest and the lowest part of the ribs margin of the median axial line [15, 16].

Serum glucose concentrations were measured using an automatized glucose oxidase method, with an overall inter-assay coefficient of variation (CV) of <5%. Serum triglyceride concentrations were measured after lipase hydrolysis in an automatic analyzer with a tungsten lamp. The inter-assay CV was 5.7%. HDL-C was measured by an enzymatic colorimetric direct method after eliminating chylomicrons, very-low-density lipoproteins (VLDL), and low-density lipoproteins by enzymatic digestion; the inter-assay CV was 5.02% [15, 16].

Details on the methodology for anthropometric, clinical and biochemical measurements, as well as the methods used to calculate the sample, have been reported in previous publications [15, 16].

The country was divided into four geographic regions for analysis. The **Northern** region, includes the states of Baja California, Baja California Sur, Coahuila, Chihuahua, Durango, Nuevo Leon, Sonora and Tamaulipas; the **Central** region, the states of Aguascalientes, Colima, Guanajuato, Jalisco, Mexico, Michoacan, Morelos, Nayarit, Queretaro, San Luis Potosi, Sinaloa and Zacatecas; the **Mexico City** region; and the **Southern** region, the states of Campeche, Chiapas, Guerrero, Hidalgo, Oaxaca, Puebla, Quintana Roo, Tabasco, Tlaxcala, Veracruz and Yucatan.

A principal component analysis of household appliances and community conditions was estimated as a proxy for socio-economic level (SEL). The variables included in the analysis were household characteristics (floor and ceiling material, total number of rooms in the household), number of persons residing in the household, possession of refrigerator, washing machine and stove and the number of electric appliances in the household (radio, TV, video player, telephone, and computer). The main first principal component explained 40.4% of the total variance with a Kaiser-Mayer-Olkin measure of sampling adequacy equal to 0.83. The range of the first principal component scores was divided into tertiles and used as a proxy for low, medium and high SEL [16].

### Diagnosis of metabolic syndrome

The diagnosis of MS was made when at least 3 components of MS were altered according to the harmonized criteria of the International Diabetes Federation (IDF), World Health Organization (WHO), National Heart, Lung and Blood Institute (NHLBI), International Atherosclerosis Society and the International Association for the Study of Obesity [4] (Table 1).

**Table 1:** **Criteria for metabolic syndrome diagnosis**

Conditions	Cutoff points
Hypertension (mm Hg) or drug treatment for hypertension control	≥130/85
Tryglicerides (mg/dL)	≥ 150
High density lipoprotein cholesterol (mg/dL)	
Men	< 40
Women	< 50
Waist Circumference (cm)	
Men	≥ 90
Women	≥ 80
Glucose (mg/dl) or drug treatment for glucose control	≥ 100

Adapted from Alberti KGMM. *et al.*[4].

(Three or more conditions).

### Metabolic syndrome groups

Combinations of MS were grouped in relation to the presence of hypertriglyceridemia and hypoalphalipoproteinemia into 4 mutually-exclusive groups: the ***mixed dyslipidemia*** group consisted of those with altered triglyceride and HDL-C levels; the ***hypoalphalipoproteinemia*** group consisted of those subjects with normal triglyceride levels but low HDL-C levels; the ***hypertriglyceridemia*** group had subjects with elevated triglyceride levels but normal HDL-C levels; and the ***no dyslipidemia*** group were subjects with normal triglyceride and HDL-C levels.

### Statistical analysis

#### Exploratory analysis

For comparison between different groups, analysis of variance was used for continuous variables and χ^2^ or difference between proportions tests were used for categorical variables. The Bonferroni correction was used in multiple comparisons [17].

#### Inferential statistics

In order to predict the group in which an individual belongs with the highest probability, an unconditional multinomial logistic regression model was fitted in which the reference MS group was the mixed dyslipidemia. The dependent variable was the categorical variable indicating the MS group to which the individual belonged and the independent variables were sex, age, geographic region, location, SEL tertile, level of education, nutritional state and previous diagnosis of diabetes and hypertension. The Hosmer-Lemeshow test was performed to assess the goodness of fit of the model [18].

Estimation procedures included sampling weights to adjust for the sample complex design. Analysis was carried out using the SVY module for complex samples with the STATA version 11.1 statistical package.

#### Ethical considerations

Because this is a secondary study, the investigators have no access to the subject’s personal information. Originally, for the ENSANUT 2006, the participants were informed about the objectives of the survey, the methodology used and consent to participate was obtained. This protocol and the ENSANUT protocol was approved by the Ethics Committee at the Mexican National Institute of Public Health.

## Results

For this study, we included cases reporting at least eight hours of fasting at the time the blood sample was collected (n = 6021). We excluded pregnant women (n = 95), and 620 cases with missing data or samples with biological implausible data for glucose (<30 mg/dl), HDL-C and triglycerides (<10 mg/dl), waist circumference (<50 cm) or blood pressure (systolic blood pressure < 80 mmHg or diastolic blood pressure < 50 mmHg) leaving a total analytic sample of 5,306 observations. An attrition analysis, based on sociodemographic variables as sex, age, SEL and residence area was carried out showing no significant differences between the subjects excluded and included.

The prevalence of MS in the Mexican population was 49.9%. The most prevalent components were low HDL-C (76.8%) and abdominal obesity (73.6%), while the least prevalent was hypertriglyceridemia (30.9%). The prevalence of hypertension, abdominal obesity and high glucose was not statistically different between urban and rural areas, by geographic region, and by SEL (*p >0.05*) (data not shown).

Seven percent of the subjects with elevated triglycerides reported to have been previously diagnosed, while more than 80% of subjects with elevated triglycerides had MS. 64.4% of subjects with abdominal obesity and 56.4% of subjects with low HDL-C had MS.

### Metabolic syndrome groups

Table 2 shows the prevalence of the MS groups and combinations among them. The MS group with hypoalphalipoproteinemia was the most prevalent in the overall Mexican population (46.0%), while the most frequent combination of MS was low HDL-C + high blood pressure (HBP) + abdominal obesity (AO) (20.0%). The same MS group and combination was observed with the highest frequency in women as in the general population, while the group of MS with mixed dyslipidemia was most prevalent in men (43.5%), with the most common combination in this group being hypertriglyceridemia (HTG) + low HDL-C + AO. The least prevalent group was MS without dyslipidemia (3.1).

**Table 2:** **Prevalence of metabolic syndrome groups and combinations stratified by sex**

Metabolic syndrome groups and combinations			Overall		Women		Men	
		Number of components altered	n = 2707		n = 1716		n = 991	
			%	95% CI	%	95% CI	%	95% CI
**Group 1: Mixed dyslipidemia**			**40.7**	**(37.5,44.1)**	**38.5**	**(34.5,42.6)**	**43.5**	**(38.9,48.2)**
Low HDL-C + High Triglycerides +	Hypertension	3	1.2	(0.7,2.1)	0.4	(0.1,1.2)	2.2	(1.2,4.0)
	High Glucose	3	1.5	(0.9,2.5)	0.6	(0.3,1.2)	2.6	(1.4,5.0)
	Abdominal Obesity	3	11.7	(9.7,14.0)	11.6	(9.3,14.4)	11.8	(8.5,16.0)
	Hypertension + High Glucose	4	0.4	(0.2,0.9)	0.1	(0.0,0.3)	0.9	(0.4,1.8)
	Hypertension + Abdominal Obesity	4	8.4	(6.7,10.4)	8.8	(6.5,11.7)	7.9	(5.9,10.5)
	High Glucose + Abdominal Obesity	4	7.6	(5.8,10.0)	8.2	(5.7,11.7)	6.9	(4.8,9.7)
	Hypertension + Abdominal Obesity + High Glucose	5	10.0	(8.3,12.1)	8.9	(7.2,10.9)	11.4	(8.5,15.1)
**Group 2: Hypoalphalipoproteinemia**			**46.0**	**(42.6,49.5)**	**51.6**	**(47.3,55.8)**	**39.2**	**(34.7,44.0)**
Low HDL-C+	Hypertension + High Glucose	3	1.4	(0.9,2.1)	0.8	(0.4,2.0)	2.0	(1.3,3.2)
	Hypertension + Abdominal Obesity	3	20.0	(17.6,22.6)	20.4	(17.5,23.6)	19.4	(15.8,23.7)
	High Glucose + Abdominal Obesity	3	11.7	(9.9,13.8)	16.2	(13.3,19.4)	6.2	(4.4,8.5)
	Hypertension + Abdominal Obesity + High Glucose	4	13.0	(11.3,14.9)	14.1	(12.0,16.6)	11.6	(9.1,14.7)
**Group 3: Hypertriglyceridemia**			**10.2**	**(8.4,12.2)**	**7.8**	**(5.9,10.2)**	**13.1**	**(10.3,16.6)**
High triglycerides+	Hypertension + High Glucose	3	0.5	(0.3,1.2)	0.1	(0.0,0.8)	1.1	(0.4,2.4)
	Hypertension + Abdominal Obesity	3	2.8	(2.0,3.8)	2.1	(1.2,3.4)	3.7	(2.5,5.6)
	High Glucose alterada + Abdominal Obesity	3	2.9	(2.1,4.0)	3.1	(2.1,4.5)	2.8	(1.6,4.8)
	Hypertension + Abdominal Obesity + High Glucose	4	3.9	(2.9,5.2)	2.5	(1.6,3.9)	5.5	(3.7,8.1)
**Group 4: Without dyslipidemia**			**3.1**	**(2.154,4.353)**	**2.2**	**(1.4,3.4)**	**4.1**	**(2.5,6.8)**
	Hypertension + Abdominal Obesity + High Glucose	3	3.1	(2.2,4.4)	2.2	(1.4,3.4)	4.1	(2.5,6.8)

#### Sociodemographic characteristics of the MS groups

The prevalence of MS was similar between men and women. However, when it was stratified by group, the hypoalphalipoproteinemia group was 31% more prevalent in women (51.5%) than men (39.2%) (*p <0.05)*, while hypertriglyceridemia group was 68% more prevalent in men (13.1%) than in women (7.8%) (*p <0.05)*.

As age increased, the prevalence of MS also increased. This prevalence was <30% in younger subjects, doubled in those older than 40 y, and was >70% in subjects over 50 y. The prevalence of the mixed dyslipidemia group was slightly higher than 40% in subjects between 20–50 y. Nevertheless, a decrease of 30.3% was observed in those ≥ 60 y.

The prevalence of MS was higher in urban areas than in rural areas (51.2% versus 45.1%, *p <0.05*). Similar results were seen in all MS groups except for the hypoalphalipoproteinemia group, which showed a 27% higher prevalence in rural than urban areas (*p <0.05)*.

The prevalence of MS was similar among the SEL tertiles. However, the hypoalphalipoproteinemia group was 22% more prevalent in the low SEL when compared to the high SEL tertile (*p <0.05)* (Table 3).

**Table 3:** **Prevalence of metabolic syndrome and metabolic syndrome groups according to sociodemographic characteristics**

			Metabolic syndrome groups			
		Metabolic syndrome (Overall)	Mixed dyslipidemia	Hypoalphalipoproteinemia	Hypertriglyceridemia	Without dyslipidemia
		n = 2707	n = 980	n = 1349	n = 285	n = 93
	n	% (95% CI)	% (95% CI)	% (95% CI)	% (95% CI)	% (95% CI)
Total n = 2707	2,707	49.9	40.7	46.0	10.2	3.1
***Sex***						
Women^a^	1,716	50.4(47.5,53.3)	38.5(34.5,42.6)	51.5(47.3,55.8)	7.8(5.9,10.2)	2.2(1.4,3.4)
Men	991	49.4(45.9,52.8)	43.5(38.9,48.2)	39.2(34.7,44.0)^a^	13.1(10.3,16.5)^a^	4.1(2.5,6.8)
***Age***						
20-29^a^	295	27.1(22.9,31.8)^b,c,d,e^	45.4(35.6,55.5)	47.0(37.6,56.6)	4.4(2.2,8.7)^d,e^	3.3(0.6,16.1)
30-39^b^	633	44.5(40.5,48.6)^a,c,d,e^	43.2(36.8,49.8)^e^	44.8(38.5,51.2)	10.6(6.8,16.0)	1.5(0.8,2.9)
40-49^c^	638	62.0(57.2,66.6)^a,b^	41.7(35.5,48.2)	48.0(41.7,54.4)^d^	7.7(5.6,10.6)^d^	2.6(1.6,4.1)
50-59^d^	509	70.0(64.9,74.7)^a,b^	44.4(38.2,50.7)^e^	36.1(30.6,42.1)^c,e^	16.2(12.1,21.3)^a,c^	3.3(1.9,5.6)
≥60^e^	632	70.3(66.2,74.0)^a,b^	30.3(25.4,35.7)^b,d^	52.9(46.6,59.1)^d^	11.7(8.2,16.4)^a^	5.1(3.1,8.2)
***Area***						
Rural^a^	774	45.1(41.2,49.1)	35.5(30.2,41.1)	55.7(50.2,61.1)	6.8(4.9,9.6)	2.0(1.2,3.2)
Urban	1,933	51.2(48.4,54.0)^a^	42.0(38.2,45.9)	43.7(39.7,47.8)^a^	11.0(8.9,13.4)^a^	3.3(2.2,4.9)
***Region***						
North^a^	714	50.7(47.3,54.2)	35.8(30.2,41.9)	49.8(43.8,55.8)^c^	9.6(6.5,13.8)^c^	4.8(3.2,7.1)^d^
Center^b^	937	50.2(45.6,54.8)	43.1(37.2,49.3)	44.9(39.0,50.9)^c,d^	8.5(6.2,11.7)^c^	3.5(1.8,6.5)
Mexico City^c^	97	56.4(44.9,67.2)	57.9(48.0,67.3)^d^	10.5(5.4,19.3)^a,b,d^	29.5(20.7,40.2)^a,b,d^	2.1(0.3,11.7)
South^d^	959	47.3(44.3,50.3)	35.7(31.2,40.4)^c^	55.9(51.0,60.7)^b,c^	6.9(5.2,9.1)^c^	1.5(0.9,2.7)^a^
***Socioeconomic Level***						
Low^a^	1,056	48.5(45.1,51.9)	38.3(33.7,43.1)	51.6(46.6,56.5)^c^	8.1(6.0,10.7)	2.0(1.3,3.2)
Middle^b^	961	51.3(48.0,54.7)	42.5(37.4,47.7)	45.1(40.2,50.1)	10.5(7.9,13.9)	1.9(1.2,3.0)
High^c^	682	50.3(45.5,55.2)	41.0(34.9,47.4)	42.1(36.1,48.4)^a^	11.5(8.2,15.9)	5.3(3.0,9.1)
***Eduaction Level***						
Less than elementary school^a^	1,739	57.4(54.7,60.1)	37.7(33.8,41.7)	49.3(45.2,53.3)	9.9(8.0,12.2)	3.2(2.3,4.4)
Elementary school or higher	965	42.9(39.5,46.4)^a^	44.8(39.3,50.3)	41.7(36.4,47.3)	10.5(7.8,14.1)	3.0(1.5,6.0)
Diabetes previously diagnosed	677	79.7(75.2,83.5)	35.9(30.5,41.6)	49.0(43.0,55.0)	11.1(7.8,15.4)	4.0(2.4,6.6)
Hypertension previously diagnosed	332	89.0(82.3,93.4)	38.5(30.7,46.9)	50.5(41.7,59.3)	9.1(5.6,14.4)	1.9(0.9,3.9)
Hypertriglyceridemia previously diagnosed	175	76.3(67.2,83.4)	42.8(33.1,53.2)	38.5(28.6,49.4)	15.5(8.7,26.1)	3.2(1.1,8.7)
**Body Mass Index Category (Kg/m** ^**2**^ **)**						
18.5 – 24.9^a^	348	23.8(20.2,27.9)^b,c^	46.0(37.5,54.8)	40.6(33.4,48.3)	10.3(6.9,15.0)	3.2(1.2,7.7)
25 – 29.9^b^	1,122	54.2(50.6,57.7)^a,c^	40.8(36.2,45.5)	45.2(40.3,50.2)	10.7(8.2,14.0)	3.3(1.7,6.2)
≥ 30^c^	1,203	73.9(70.7,76.9)^a,b^	38.4(33.6,43.4)	49.2(44.3,54.2)	9.6(7.3,12.6)	2.8(1.9,3.9)

^a, b, c ,d, e^ *P Value <0.05* for difference between proportions when comparing between categories of sociodemographic characteristics, with Bonferroni Adjustment.

The predictors associated with belonging to the hypoalphalipoproteinemia group in the multinomial logistic regression model were being female and living in the northern region of the country when compared to living in Mexico City (*p <0.01)*. The factors that best predicted belonging to the hypertriglyceridemia group were being male, having over 50 y and residing in Mexico City when compared to the northern region (*p <0.01)*. Finally, there was a lower probability of finding individuals without dyslipidemia in the southern region of the country (*p <0.05)* (Table 4).

**Table 4:** **Sociodemographic predictors of metabolic syndrome groups**

	Mixed dyslipidemia	Hypoalphalipoproteinemia		Hypertrigliceridemia		Without dyslipidemia	
	OR (95% CI)	OR (95% CI)		OR (95% CI)		OR (95% CI)	
*Sex (women)*							
Men	1.0	0.69**	(0.53 , 0.91)	1.71**	(1.14 , 2.55)	1.61	(0.93 , 2.77)
*Age (20–29 years)*							
30-39 years	1.0	0.99	(0.60 , 1.63)	2.71*	(1.09 , 6.74)	0.39	(0.06 , 2.65)
40-49 years	1.0	0.99	(0.62 , 1.59)	1.94	(0.79 , 4.72)	0.86	(0.15 , 4.97)
50-59 years	1.0	0.70	(0.42 , 1.16)	4.31**	(1.74 , 10.70)	1.05	(0.15 , 7.10)
≥ 60 years	1.0	1.51	(0.91 , 2.48)	4.36**	(1.63 , 11.71)	2.47	(0.49 , 12.29)
*Area (Rural)*							
Urban	1.0	0.83	(0.61 , 1.14)	1.07	(0.64 , 1.79)	1.11	(0.55 , 2.22)
*Region (North)*							
Central	1.0	0.78	(0.53 , 1.13)	0.73	(0.40 , 1.34)	0.69	(0.30 , 1.59)
Federal District	1.0	0.12**	(0.05 , 0.27)	1.92*	(1.01 , 3.67)	0.27	(0.04 , 1.95)
South	1.0	1.07	(0.75 , 1.54)	0.79	(0.44 , 1.39)	0.43*	(0.19 , 0.99)
*Socioeconomic Level (Low)*							
Middle	1.0	0.92	(0.69 , 1.24)	1.11	(0.67 , 1.83)	0.80	(0.38 , 1.68)
High	1.0	0.99	(0.68 , 1.44)	1.19	(0.65 , 2.19)	2.29	(0.88 , 5.91)
*Education Level (Less than primary)*							
Primary or higher	1.0	0.93	(0.68 , 1.28)	0.96	(0.61 , 1.54)	0.73	(0.39 , 1.37)
*Body mass Index Category (Normal)*							
Overweight	1.0	1.37	(0.89 , 2.12)	1.04	(0.57 , 1.89)	1.27	(0.33 , 4.83)
Obesity	1.0	1.5	(0.97 , 2.31)	1.14	(0.62 , 2.09)	1.20	(0.35 , 4.07)
Diabetes previously diagnosed	1.0	1.17	(0.78 , 1.76)	0.71	(0.40 , 1.26)	0.47	(0.19 , 1.14)
Hypertension previously diagnosed	1.0	1.24	(0.90 , 1.70)	1.03	(0.63 , 1.69)	1.26	(0.64 , 2.50)

***p Value <0.01, Multinomial regression Model. Reference group: Mixed Dyslipidemia.

**P Value <0.05, Multinomial regression Model. Reference group: Mixed Dyslipidemia.

## Discussion

This study shows different MS groups as well as the prevalence of these groups according to sociodemographic variables. We classified four mutually exclusive groups of MS based on the absence or presence of changes in triglycerides and HDL-C since these factors play an important role in the development of MS, insulin resistance, and because they are major mediators in the atherogenic process. In addition, several studies have identified the lipid profile as an insulin resistance and cardiovascular risk marker [11, 12, 19].

The prevalence of MS in our study was 49.9%, only a 0.1% point higher than the one previously reported [13]. This difference is explained by our selection criteria where cases with at least one missing value in any MS component were excluded from the analytic sample.

While MS appears to be characteristic of certain population groups such as older subjects and those living in urban areas, a similar distribution was observed by SEL tertile. When MS was stratified by group, the following characteristics were observed:

### Mixed dyslipidemia group

The principal finding in this group was the decreased prevalence in subjects ≥60 y, which may be explained by a higher risk of mortality in the combined presence of low HDL-C and hypertriglyceridemia. Mazza *et al.* followed 3,257 women for 12 years and found that those in the lowest HDL-C quintile and the highest triglycerides quintile had a mortality risk almost three times higher than women in the highest HDL-C quintile or those in the lowest triglycerides quintile [11].

### Hypertriglyceridemia group

This group was more prevalent in men, in the Mexico City region and in subjects ≥50 y. It has been reported that a high consumption of alcoholic beverages and simple carbohydrates increase the risk of hypertriglyceridemia. A report of the ENSANUT 2006 revealed that consumption of alcoholic and sugar sweetened beverages was higher in men than in women, with the latter being the primary source of simple sugars in the Mexican diet [20, 21].

The fact that this group was more prevalent in Mexico City may be explained by the lifestyle of its inhabitants, which include a higher consumption of industrialized food, and lower physical activity levels compared to the rest of the regions [22, 23]. However, these results should be interpreted with caution since the sample size in this region was small when compared to other regions.

### Hypoalphalipoproteinemia group

The hypoalphalipoproteinemia group was more prevalent in the south region, low SEL and rural areas. High total fat, mono and polyunsaturated fatty acids consumption increases HDL-C concentrations [24, 25]. Therefore, differences in dietary patterns among regions of the country could partially explain variations in MS groups. Studies of ENSANUT, that evaluated the distribution of fat consumption by sociodemographic characteristics, found that around 60% have a low consumption of polyunsaturated fats, and a lower total fat and mono- and polyunsaturated fat consumption was found in rural locations, in the southern region of the country and in subjects in the lowest SEL [21, 26]. Another possible explanation for low HDL-C and normal triglycerides is the higher prevalence of physical activity in this population given that physical activity has been associated with an increase in lipoprotein lipase activity, which produces a decrease in triglyceride levels [27]. According to ENSANUT 2006, the prevalence of physical activity was higher in these locations [22].

The hypoalphalipoproteinemia group was more prevalent in women when compared to men. The difference in prevalence between men and women in the MS groups with normal HDL-C may be explained by the different cutoff points for hypoalphalipoproteinemia between genders. The harmonized MS criteria and the ADA proposed different cutoff points for HDL-C between men and women (<40 mg/dl in men; <50 mg/dl in women) [4, 28]. These differences have been proposed because the average HDL-C levels are higher in women than in men, and having the same cutoff point would diagnose more men than women. Nevertheless, it does not necessarily mean that the risk is different [29]. HDL-C levels according to gender have been studied in other populations; Davis *et al.* evaluated mean HDL-C levels in men and women in six countries and found that women had higher HDL-C levels on average. However, there was great variability found among populations. The highest difference found between men and women (15.6 mg/dl) was reported in Canada while a smaller difference (2.3 mg/dl) was found in China [30]. Differences between men and women Korean adults were similar than those in the Chinese population (2.5 mg/dl) [31].

In our population, the age-adjusted difference in HDL-C by gender was 3.2 mg/dl (38.9 mg/dl in women and 35.7 mg/dl in men), similar to that seen in the Chinese and Korean populations. When the 40 and 50 mg/dl cutoff points were used for men and women respectively, the prevalence of hypoalphalipoproteinemia was 68.7% for men and 83.6% for women, but when the 40 mg/dl cutoff point was used for both genders, the prevalence of hypoalphalipoproteinemia in women decreased by 27.6 percentage points and the prevalence was even higher in men than in women (68.7% versus 56.0%). In addition, the prevalence of MS in women decreased from 50.4% to 42.4% and the prevalence of MS with normal HDL-C (hypertriglyceridemia and no dyslipidemia groups) increased from 10% to 29%.

The use of different criteria for hypoalphalipoproteinemia by gender in the Mexican population does not appear to be completely justified given the similarity in HDL-C levels by gender. When these criteria are applied, the prevalence of MS and its groups may be differentially affected. Given that the cutoff points of 40 mg/dl and 50 mg/dl were made based on the large HDL-C differences found between genders in other populations, it may be advisable to rethink these cutoff points for the Mexican population as well as for other populations in which the difference in HDL-C by gender is very small, as seen in the Korean and Chinese populations [30, 31].

### Group without dyslipidemia

Unlike the hypoalphalipoproteinemia group, the group without dyslipidemia was uncommon in the south region of the country and in the low socioeconomic level group. This may also be explained by the quality of diet in this population [21].

One of the most interesting results found in this study is the low level of medical diagnoses of hypertriglyceridemia. Nevertheless, 80% of the subjects with elevated triglyceride levels had MS. Thus, it may be advisable to include serum triglyceride measurements in routine medical evaluations, as this is an area of opportunity for preventing cardiovascular events given that hypertriglyceridemia is a modifiable risk factor.

One of the limitations of this study is that there was not sufficient power to include more analytical variables such as dietary and physical activity characteristics that may contribute to explain the sociodemographic differences between the different MS groups. Given that this is a cross-sectional analysis derived from a national survey in which additional measurements of cardiovascular risk were not collected, it was not possible to identify groups or combinations with a higher cardiovascular risk.

There is no general agreement regarding an adequate waist circumference cutoff point for the Mexican population due to significant differences in height and body composition compared to Caucasians and other ethnic groups. However, the latest IDF MS definition recommends a lower cutoff point for central and South American populations [4]. Besides, a major sensitivity analysis in Mexican population showed that the IDF cutoff point had a better sensitivity for detection of diabetes, hypertension and metabolic syndrome, compared with the AHA cutoff point [32].

Other authors have described groups based on different MS components such as abdominal obesity [33]. However the MS groups based on lipid alterations seem to be useful because in our population 95% of the subjects with MS have abdominal obesity, therefore grouping MS using this factor does not provide balanced groups useful to characterizing the condition. In addition, the distribution of the other components (high glucose, hypertension and abdominal obesity) in the subjects with MS, among area, geographic region and SEL was not significantly different. A factor analysis of information coming from African population showed that the strongest predictors for MS were high blood pressure, triglycerides and HDL-C, which could support our MS classification [34].

## Conclusions

The MS classification proposed here could be useful to describe different characteristics of this condition. From a public health perspective, this approach could be helpful to identify and to target prevention, treatment and early detection interventions according to demographic and biological characteristics. For example, for the population living in the south of Mexico in rural areas and with low SEL, an intervention could be focused on increasing the consumption of mono and polyunsaturated fatty acids while in the central region of the country and Mexico City the promotion of physical activity could be more important given the most prevalent groups in those regions (hypoalphalipoproteinemia group and hypertriglyceridemia group respectively).

This is the first study in Mexico to describe and classify groups of MS combinations. Subsequent analyses could evaluate these groups to better understand the different cardiovascular and metabolic outcomes associated.
